# Carbon stock under major land use/land cover types of Hades sub-watershed, eastern Ethiopia

**DOI:** 10.1186/s13021-019-0122-z

**Published:** 2019-05-22

**Authors:** Tessema Toru, Kibebew Kibret

**Affiliations:** School of Natural Resources Management and Environmental Sciences, College of Agriculture and Environmental Sciences, Haramaya University, Dire Dawa, Ethiopia

**Keywords:** Carbon sequestration, Carbon, Climate change, Degradation, Deforestation, Land use

## Abstract

**Background:**

Developing land management scenarios that have the potential to sequester carbon and reduce greenhouse gasses (GHG) emission on a sustainable basis entails quantifying the current carbon stock under different land uses. In light of this, a study was conducted in Hades sub-watershed, eastern Ethiopia, to explore the carbon stock under four major land uses: natural forest, coffee agroforestry, grazing land and cropland, involving samples from four carbon pools: aboveground, belowground, litter, and soil. To this end, vegetation and soil samples were collected from the respective land uses following recommended procedures.

**Results:**

The results indicate that organic carbon concentration decreased with soil depth though substantial amount of carbon was found in the lower soil depths under land use with woody perennials. The mean total organic carbon stock ranged from 138.95 t ha^−1^ in the cropland to 496.26 t ha^−1^ in the natural forest. The soil organic carbon stock was found to be relatively higher than that of the vegetation carbon stock in the natural forest and coffee agroforestry land uses.

**Conclusion:**

This study highlights the importance of assessing watershed level carbon stock for better and carbon friendly land use decision making. Land uses with woody perennials have high carbon stock than those without. Hence, conservation-based production systems with inclusion of woody perennials are options suggested to enhance carbon sequestration in the sub watershed.

## Background

The prominent factor deriving climate change is the increase in the concentration of greenhouse gases (GHGs) in the atmosphere. The ever increasing concentrations of carbon dioxide (CO_2_), methane (CH_4_), nitrous oxide (N_2_O), and other GHGs have distorted the balance between the incoming and the outgoing solar radiation emitted by the Sun and Earth, respectively [1]. IPCC [2] estimated the increase in global temperature because of radiative forcing of GHGs in the atmosphere at 0.6 °C in the 1990s, while highlighting that this is expected to increase by 1.4 to 5.8 °C by 2100. Carbon dioxide, the second most important GHG, alone has increased from its pre-industrial 280 ppm to the current 397 ppm (International Energy Agency [3].

The two most common factors distorting the carbon cycle are land use change and combustion of fossil fuel [4]. Practically, about 75% of the global CO_2_ emissions result from the combustion of fossil fuels in transportation, in building heating and cooling, and in the manufacture of cement and other goods [5]. In Ethiopia, the emission from fossil fuel generated 2.3 million tons of CO_2_ in 1990, and the figure increased to 8.5 million tons of CO_2_ in 2013 [3].

Land use change, especially conversion of a natural system into a managed system, results in alteration of the carbon balance [6]. There is also tremendous evidence showing the negative impact of agriculture on carbon stock [7–11]. However, agriculture is among the land use practices that emit as well as sequester CO_2_. It may lose soil organic matter due to intense decomposition following soil plowing, removal of above ground biomass during harvest, and severe soil erosion inherent in these activities [7]. Nevertheless, if proper land use and management practices are put in place, agriculture can serve as an important sink [12–15].

In the tropics, deforestation is the second most important source of greenhouse gases (GHG) after fossil fuel combustion [16]. Conversion of forest land to other land uses, such as agriculture, enhances decomposition and removal of carbon through harvest [8, 9, 17–20]. On the contrary, a significant increase (50%) of soil carbon was reported after the conversion of arable land into forest land [21, 22].

The positive effects of other land uses, such as agroforestry, on carbon balance were also highlighted. In soils that were previously under cultivation, agroforestry systems were found to be highly effective in restoring soil carbon [23]. Similarly, higher aboveground carbon in coffee agroforestry (61.5 ± 25.0 t ha^−1^) than in woodland, pasture, and cropland, but slightly less than that in natural forest (82.0 ± 32.1 t ha^−1^) was reported in south western Ethiopia [24]. The same study further indicated that about 59.5 t ha^−1^ organic carbon could have been lost if the coffee agroforestry had been converted into cropland.

Similar to the other land uses, rangelands and grazing areas are storing carbon above and below ground. In Europe, an increase in carbon stock following conversion of cropland into grass land was reported [25]. Though there is scanty information on carbon stock of grazing lands in highland areas of Ethiopia, 128.39 t ha^−1^ belowground (soil and root) and 13.11 t ha^−1^ above ground, organic carbon was reported in the communally managed semi-arid rangelands in southern Ethiopia [26].

Hence, understanding the relationship between land-use systems and carbon stock is essential, as every land use system has either positive or negative impact on the carbon balance. Besides, considering the potential and constraints of a watershed in relation to carbon stock is vital to set recommendation on the maintenance and enhancement of carbon stock. In Ethiopia, few area-specific researches have been conducted on organic carbon stock and sequestration. The available researches are limited in their scope, mostly concentrating on soil carbon and giving much less emphasis to the carbon stock of the various carbon pools at watershed level. Furthermore, the impact, on carbon stock, of smallholder subsistence farming, mostly characterized by low level of management where limited or no inputs are used, under high population pressure has not been studied comprehensively. Under the looming climate change, such information on carbon emission and sequestration is essential for developing strategies that enhance productivity (through increasing carbon stock) and abate greenhouse gas emissions (through enhancing carbon sequestration). In view of this backdrop, this study assessed the current carbon stock under different land use types and carbon pools in Hades sub-watershed, eastern Ethiopia.

## Methods

### Description of study area

**Fig. 1:**
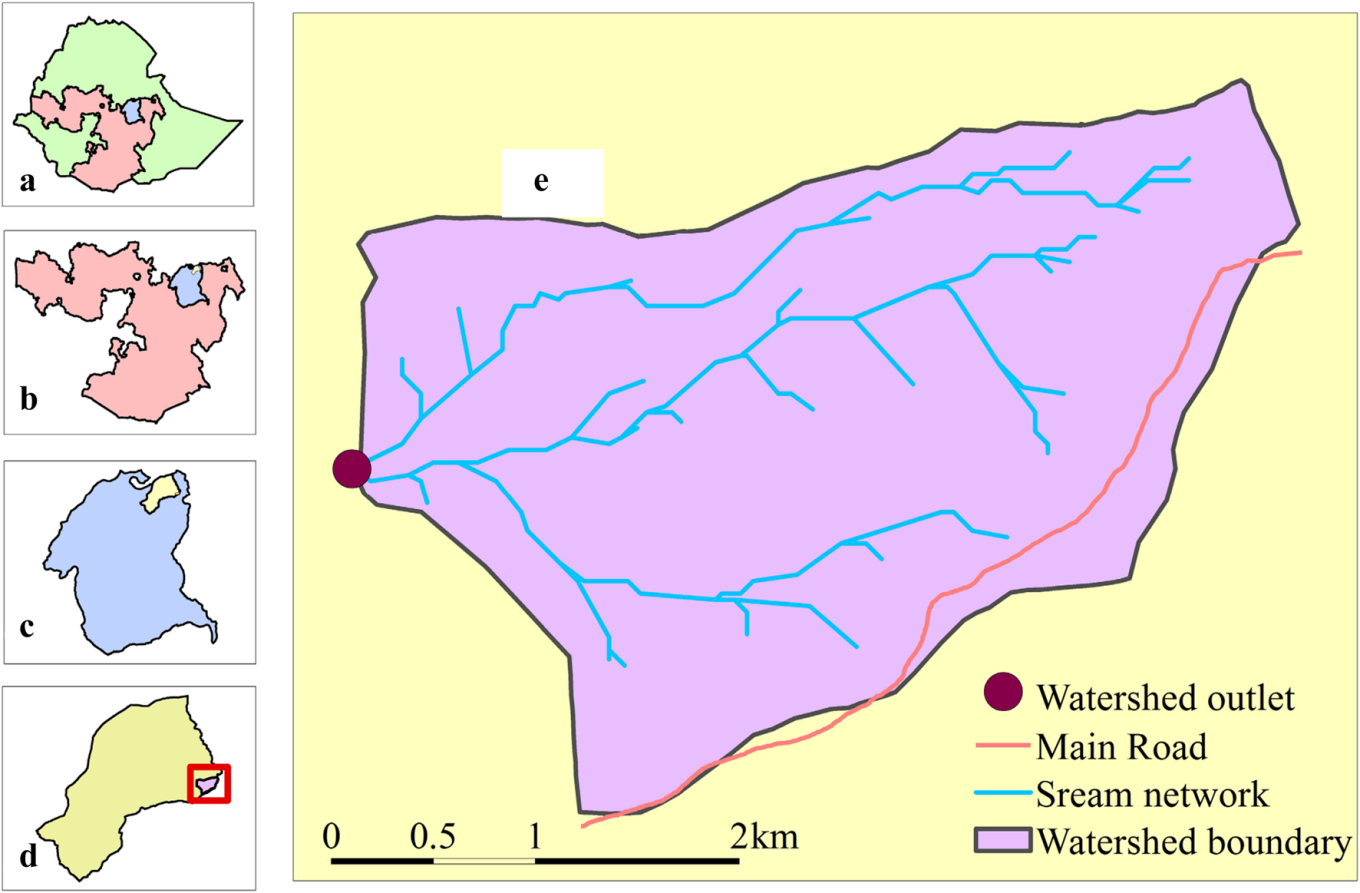
Location of the study site **a** Ethiopia; **b** Oromiya Region; **c** Western Hararghe; **d** Doba Woreda (District); and **e** the study area

The study was conducted on major land uses of Hades sub-watershed in eastern Ethiopia. It is located at about 401 km away from Addis Ababa along the highway to Dire Dawa and Harar cities. The geographical location of the sub-watershed is 9°12′0″9°19′0′′ N and 41°15′0′′–41°17′0′′ E (Fig. 1). The altitude of the study area ranges from 1995 to 2775 m.a.s.l. The precipitation of the area is characterized by bi-modal distribution with mean annual rainfall of 930 mm. The mean annual temperature of the area ranges from 10.3 to 18.9 °C. The Hades forest used to cover most of the present crop and grass lands and coffee agroforestry sites. Currently, the forest is degraded due to continuous disturbance. The remaining dominant tree species are *Afrocarpus gracilior*, *Croton macrostachyus*, *Hagenia abyssinica*, *Schefflera abyssinica*, and *Prunus africana.* The farming practice in the area is mixed farming whereby the community cultivates annual and perennial crops and manages livestock. The dominant agricultural crops are sorghum (*Sorghum bicolor*), maize (*Zea mays*), wheat (*Triticum aestivum*), barley (*Hordeum vulgare*), and beans (*Phaseolus vulgaris*). Soil bunds are constructed on croplands and coffee agroforestry in order to abate soil erosion by water. According to small-scale studies carried out by [27], the dominant soil types are Leptosols.

### Delineation of major land uses of the study area

Candidate land use/land cover (LULC) categories were identified using topographic map (1:50,000) obtained from Ethiopian Mapping Authority and Google Earth online imagery and Digital Elevation Model (DEM: 30 m × 30 m resolution). Following this, physical observation was made to confirm the basic information about the major land use types and topographic variations of the study area. The boundary of the sub-watershed was delineated using GPS recordings. The LULC map of the study period was produced in ArcGIS 10.5 software. Accordingly, four major land use types (cropland, grazing land, coffee agroforestry, and natural forest) were identified (Table 1).

**Table 1:** Description of land uses in Hades sub-watershed

Land use	Symbol	Definition
Cropland	CL	Cultivated land used for production of cereal crops. In this land use, the crop residue is collected, pilled, and used for animal feed, fuel, and, in some cases, for house construction and as source of cash
Grazing land	GL	A land commonly used for open grazing and in some cases for cut and carry system. It is found scattered within the sub-watershed particularly in areas with impeded drainage
Coffee agroforestry	CA	A land use characterized by presence of coffee under *Cordia africana* as shade tree with well-built soil conservation structure and frequently enriched with partially decomposed organic matter such as cow dung
Natural forest	NF	Land covered with naturally grown trees and dominated by indigenous tree species that have reached climax whereby the understory vegetation is suppressed by the shade effect of the big trees. This land use is found mainly in the higher altitudes and steep slopes of the sub-watershed

### Sampling techniques

**Fig. 2:**
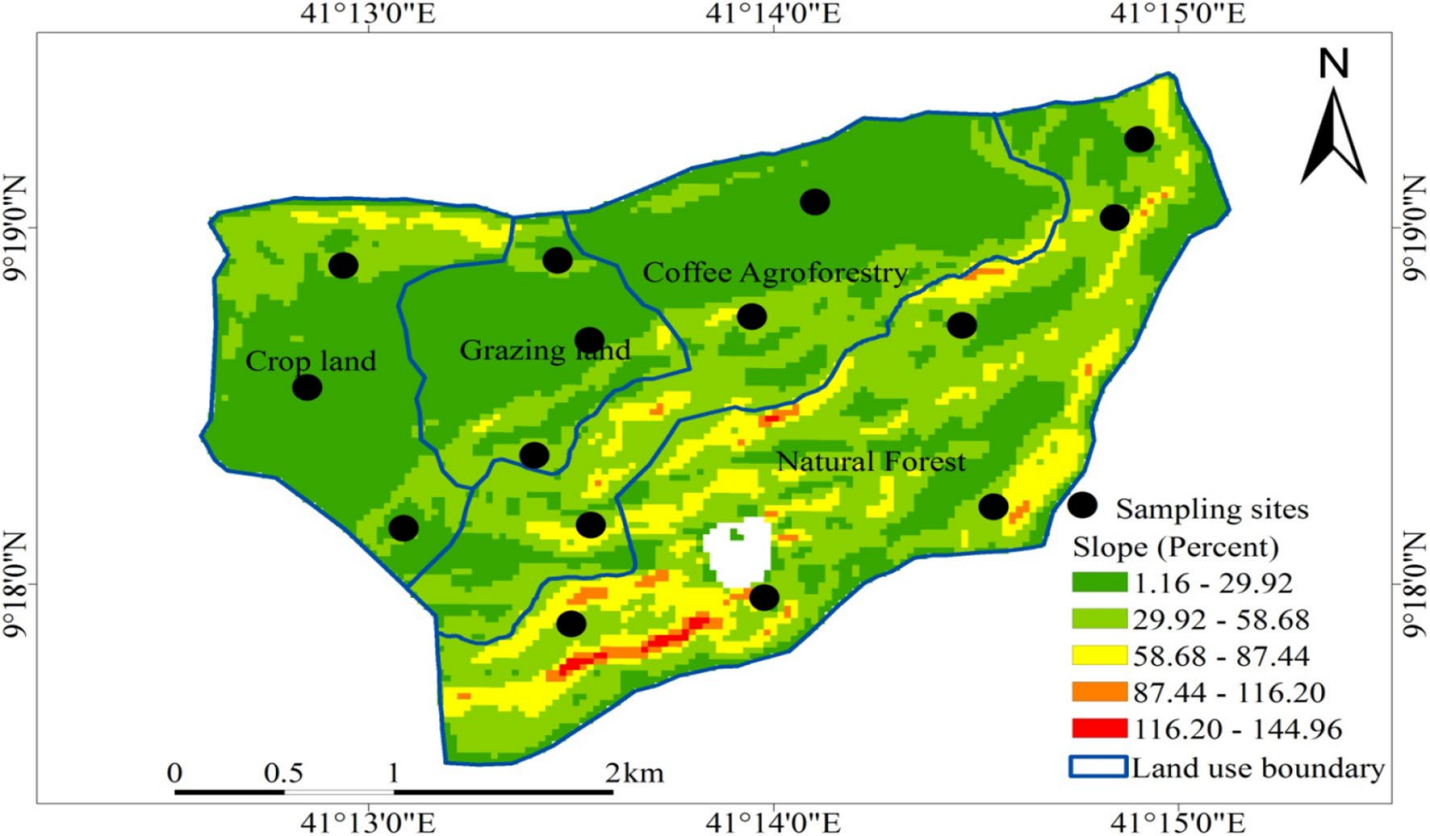
Hades subwatershed sampling sites and slope (in percent) map

Sampling sites for sample collection were the strata that were determined based on the land use/land cover types. Two transects were established along the slope at 500 m interval crossing cropland, grazing land, coffee agroforestry, and natural forest guided by compass. Besides, random sample points were established in areas not covered by the transect (Fig. 2). On crop and grass lands, sample plots of 10 m × 10 m were laid at 500 m interval. On coffee agroforestry, sample plots of 20 m × 20 m were used. A ‘nested’ sampling approach [28, 29] was used for collecting both vegetation and soil samples in the natural forest. To avoid boundary effect, the first plot was established 150 m inside the forest to the border line. The location of each sampling points were recorded using GPS.

In the sample plot, all living trees and shrubs with diameters at breast height (dbh) of ≥ 5 cm were inventorized. The dbh of the trees and shrubs were measured using diameter measuring tape [30]. All the litter samples in a 0.5 m × 0.5 m quadrat from the four corners and the center of 40 m × 5 m plot were collected and a composite sample was made. The aboveground carbon stock of coffee shrubs was estimated from the diameter measured at 40 cm from the ground using caliper [31]. As suggested by [32], the belowground biomass (root) of a plant was calculated from the aboveground biomass. The sites that were used for aboveground carbon stock measurement were also used for belowground carbon stock measurement in both the natural forest and the coffee agroforestry.

Soil samples were taken from the four corners and center of the 40 m × 5 m sub plot for the natural forest and the 10 m × 10 m plot of the crop land, grazing land, and coffee agroforestry. The samples were taken from three depths (0–20, 20–40, and 40–60 cm) using auger [33] taking into account the current standard depth of 30 cm recommended for C accounting purposes [34–36]. Hence, three composite samples per plot were collected for laboratory analysis of organic carbon, total nitrogen, soil texture, and pH. For bulk density determination, undisturbed soil samples were collected from the same depths using core sampler and from the center of the respective plots. All disturbed soil samples were air-dried, grinded, passed through a 2 mm mesh sieve for determination of the selected soil properties except organic carbon and total nitrogen in which case the samples were crashed further to pass through a 0.5 mm mesh sieve.

### Laboratory analysis

For litter carbon assessment, a sub sample of 100 g from a composite sample was oven dried at 105 °C until a constant weight was attained. Then, a sample of 4 g was ignited in Muffle furnace (Carbolite Aston-Lan. Hope, England) at 500 °C for 8 h [37]. For all the samples, the determination was made in triplicate.

Soil texture was determined by the Bouyoucos hydrometer method as described in [38]. The dry bulk density of the soils was determined using the core method as described in [39] in which case the core samples were dried in an oven set at a temperature of 105 °C to a constant weight. The bulk density was obtained by dividing the oven dry weight by the volume of the cores as is indicated in Equation [37]. Soil pH in water was measured in soil–water (1:2.5) suspension using pH meter [40]. The soil organic carbon content was determined following the Walkley–Black oxidation method [41]. Total nitrogen was determined by the micro-Kjeldahl digestion, distillation, and titration method [42].

### Biomass carbon estimation

#### Estimation of vegetation biomass and carbon

Species specific allometric equations were not available for all trees in the study area. Therefore, the dry weight biomass of each tree was calculated using the following formula [43]:

1 $$AGB = 34.4703 - 8.067D + 0.6589D^{2}$$

where AGB is aboveground biomass in kg, D is diameter at breast height (dbh).

As described by [44], root biomass in ecosystems is often estimated from root to shoot ratios. The ratio ranges from 0.18 to 0.30, with tropical forests in the lower range. As suggested by [32], the belowground biomass (root) of a plant is close to 20 percent of its total aboveground biomass. Accordingly, the root biomass of trees was estimated using the following formula:

2 $${\text{Root}}\;{\text{biomass}} = {\text{AGB}}*0.2$$

The corresponding carbon for above and below ground living biomass was obtained by multiplying the biomass with 0.5 [45].

Litter (undecomposed and unburned fallen leaves, twigs, and branches) was collected from the natural forest and coffee agroforestry, while undergrowth vegetation (all living seedling, sapling, and herbaceous species) was only collected from the natural forest. Litter and undergrowth vegetation samples were collected from 0.5 m × 0.5 m quadrants for natural forest and 10 m × 10 m plots for coffee agroforestry. The biomass and carbon stock in litter and undergrowth were calculated using the following equations [28]:

3 $$Total\;dry\;weight = \frac{Total\;fresh\;weight\;(kg) \times subsample\;dry\;weight\;(g)}{{Subsample\;fresh\;weight\;(g) \times sample\;area\;(m^{2} )}}$$

Similarly, the organic matter content of the sample was derived from the following relationship:

4 $$\% OM = \frac{{\left( {sample + CrucibleWt} \right) - \left( {Sampleafterash + CrucibleWt.} \right)}}{Sample + CrucibleWt.} \times 100$$

5 $$\% OC = \frac{\% OM}{1.742}$$

where OM is organic matter, OC is organic carbon, and 1.724 is Van Bemmelen factor [46].

The following formula was used to calculate the biomass of the coffee shrub [31]:

6 $${\text{Y}} = {\text{b}}_{1} {\text{d}}_{40}^{2}$$

where Y is the biomass, d_40_ is the stump diameter at 40 cm height and b_1_ is the coefficient for the squared power equation for the above formula produced with the cross-validation ‘‘training’’ coffee plants in Ethiopia and whose value is 0.147 [31].

The carbon stock of the shade trees in coffee agroforestry was determined using the formula developed by [30]:

7 $$AGB = 0.2035*DBH^{23196}$$

#### Estimation of soil carbon stock

The organic carbon content of soils obtained from laboratory analysis was used to calculate carbon stock per unit area of land. The carbon stock for each layer per hectare was calculated as [47]:

8 $${\text{C}}_{\text{i}} \,({\text{t}}/{\text{ha}}) = {\text{BD}}_{\text{i}} \left( {1 - {\text{CF}}_{\text{i}} } \right) \times {\text{d}}_{\text{i}} \times {\text{OC}}_{\text{i}} $$

where C_*i*_ is C stock of the ith layer in t/ha, BD_*i*_ is bulk density of the ith layer in kg/m^3^, CF_*i*_ is coarse fragment content of the ith layer, OC is the soil content of organic C in %, d_*i*_ is thickness of the ith layer (m), 10 is conversion factor from kg/m^2^ to t/ha.

Coarse fraction was determined during sample preparation after repeated crushing of clods by hand, mechanical grinding, drying, and sieving until the sample was passed through a 2 mm sieve. Following this, the coarse fraction was weighed and its proportion was determined using the relationship [47]:

9 $${\text{Coarse}}\;{\text{fraction}}\;(\% ) = \left( {\frac{{{\text{Total}}\;{\text{weight}} - {\text{weight}}\;{\text{of}}\;{\text{fraction}} < 2\;{\text{mm}}}}{{{\text{Total}}\;{\text{weight}}}}} \right) \times 100.$$

The total carbon stock for the 0–60 cm depth was calculated as:

10 $$C_{total} \left( {\text{t/ha}} \right) = \sum\limits_{i = 1}^{3} {C_{i} }$$

The dry bulk density was calculated using the following equation:

11 $${\text{BD}}\,\;\left( {{\text{kg}}/{\text{m}}^{3} } \right) = 1000 \times \frac{{{\text{M}}_{\text{ODS}}\,({\text{g}})}}{{{\text{V}}_{\text{t}}\,({\text{cm}}^{3} )}}$$

where M_ODS_ is the mass of the oven-dry soil (g); V_t_ is the total volume of the soil core calculated from:

$${\text{V}}_{\text{t}} =\uppi{\text{r}}^{2} {\text{h}}$$

where r is the internal radius of the cores measured using a caliber (cm), and h is height of the cores measured using a hand tape. 1000 is conversion factor from g/cm^3^ to kg/m^3^. π is a constant which is equal to 22/7.

The total carbon stock (ton/ha) of each land use of the sub-watershed was obtained from:

12 $$C_{totalstock} = C_{AB} + C_{BG} + C_{LT} + C_{Soil}$$

where C_AG_ is aboveground carbon, C_BG_ is belowground carbon, C_LT_ is litter carbon, C_UG_ is undergrowth vegetation carbon, and C_soil_ is soil carbon.

#### Calculation of deterioration index

Deterioration index (DI) of soils under coffee agroforestry, grazing land, and crop land was computed assuming that the level of organic carbon and total nitrogen contents under these land uses before conversion were once the same with soils under less influenced natural forest. The changes were averaged across the two dynamic soil properties to generate the overall soil deterioration index [48]. The following relationship was employed to compute deterioration index for the individual soil properties:

13 $${\text{DI}}\left( \% \right){ = }\left[ {\frac{{{\text{PSL}} - {\text{PRL}}}}{\text{PRL}}} \right] \times 100$$

where PSL is mean value of individual soil property (P) under specific land use (SL), PRL is mean value of individual soil property (P) under reference land use (RL), and DI is deterioration index.

### Statistical analysis

The data were grouped and summarized by land use and soil depth. The standard error of mean was calculated for each parameter, and depths of the soil and vegetation. The data for individual depths were also subjected to ANOVA (analysis of variance). Least significant difference (LSD) was used to separate means that are significantly different from each other at p < 0.05. Besides, statistical differences were tested using two-way analysis of variance (ANOVA) to identify whether differences, in soil attributes, by land use and depth are significant following the general linear model (GLM) procedure of SPSS Version 20.0 for Windows.

## Results

### Selected soil properties

#### Physical properties

The measured soil physical properties indicated significant (p < 0.05) differences in bulk density by land use and soil depth (Table 2). However, the interaction effect of land use by soil depth on bulk density was not significant (p ≥ 0.05) (Table 2). Across the three soil depths, significantly lower bulk density values were recorded in soils under the natural forest, whilst higher values were observed in soils under the crop land (Table 3). The other two land uses had intermediate values. Except in soils of the coffee agroforestry land use type, bulk density values increased down soil depth.

**Table 2:** Two way analysis of variance for bulk density (g/cm³), sand (%), clay (%) and silt (%) under different land uses, soil depth and interaction effect in Hades sub watershed

Source of variation	df	BD		Sand		Clay		Silt	
		MS	Sig.	MS	Sig.	MS	Sig.	MS	Sig.
Depth	2	5.683	0.002*	25.764	0.637	178.67	0.058	77.154	0.081
Land use	3	26.79	0.000*	349.681	0.002*	835.49	0.000*	190.57	0.001*
Depth × land use	6	0.897	0.339	75.083	0.271	16.237	0.941	26.467	0.487

*The mean difference is significant at the 0.05 level

**Table 3:** Contents of some selected soil physical properties in relation to different land uses and soil depths (mean ± SD)

Variable	Depth (m)	Land uses				
		NF	CA	GL	CR	Overall
Bulk density (g/cm³)	0–20	0.92 (± 0.2)	1.39 (± 0.12)	1.13 (± 0.18)	1.42 (± 0.13)	1.15 (± 0.31)a
	20–40	1.09 (± 0.11)	1.36 (± 0.17)	1.24 (± 0.09)	1.49 (± 0.13)	1.28 (± 0.21)ab
	40–60	1.40 (± 0.21)	1.53 (± 0.29)	1.27 (± 0.1)	1.61 (± 0.12)	1.48 (± 0.26)b
	Overall	1.2 (± 0.37)a	1.43 (± 0.19)ab	1.21 (± 0.13)ab	1.50 (± 0.13)b	
Sand (%)	0–20	67.83 (± 7.7)	58.67 (± 3.21)	49.67 ± 2.08	55.67 ± 12.66	59.93 (± 9.99)
	20–40	62.83 (± 2.32)	55.33 (± 7.1)	48 ± 2.1	62.33 ± 13.01	58.31 (± 8.64)
	40–60	59 (± 3.46)	58.33 (± 11.02)	54.33 ± 5.03	49 ± 3.61	56.13 (± 8.10)
	Overall	63.39 (± 7.48)b	57.44 (± 6.93)ab	50.73 (± 4.02)a	55.67 (± 10.92)ab	
Clay (%)	0–20	10.17 (± 2.75)	17 ± 6.1	29 ± 6	29.33 ± 8.1	19.13 (± 10.17)
	20–40	17.5 (± 8.88)	21 ± 7.94	35 ± 5.29	29.33 ± 7.37	24.07 (± 10.26)
	40–60	20.17 (± 2.02)	23 ± 12.49	34 ± 4.58	37 ± 5.29	26.87 (± 10.61)
	Overall	15.94 (± 8.53)a	20.33 (± 8.43)a	32.67 (± 5.39)b	31.89 (± 7.18)b	
Silt (%)	0–20	22 (± 5.29)	24.33 ± 5.03	21.33 ± 4.04	15 ± 5	20.93 (± 5.93)
	20–40	19.67 (± 7.91)	23.67 ± 1.53	16.8 ± 6.84	8.33 ± 7.1	17.63 (± 8.26)
	40–60	40.67 ± 2.89	18.67 ± 2.31	11.67 ± 1.53	14 ± 1.73	17.00 (± 3.98)
	Overall	20.67 (± 5.96)b	22.22 (± 3.93)b	16.60 (± 5.82)ab	12.44 (± 5.41)a	

*TN* total nitrogen, *NF* natural forest, *CF* coffee agroforestry, *GL* grazing land, *CR* cropland

For each parameter, different letters indicate significant differences between treatment means within one depth (p < 0.05)

Sand, silt, and clay content, on the other hand, were significantly (p < 0.05) affected by land use alone (Table 2). Accordingly, significantly higher sand content was measured in soils of the natural forest across the three depths. On the contrary, significantly higher clay content was recorded at 0–20 cm soil depth of the cultivated and grazing lands (Table 3). Except in soils of the natural forest where it decreased, sand content did not show any consistent trend with soil depth in the other land use types. Silt content, on the other hand, exhibited a downward trend with soil depth in soils of the cultivated and grazing lands, showing no consistent variation with depth in the other land use types. Owing to some downward translocation, the clay content showed an upward trend with soil depth in all land uses but the grazing land, where it followed no consistent pattern.

#### Chemical properties

The two way analysis of variance (Table 4) indicates that soil organic carbon and total nitrogen were significantly (p < 0.05) affected by soil depth and land use while pH was only significantly affected by land use (p < 0.05). These three soil properties, however, were not significantly affected by the interaction of land use and depth. On the contrary, C:N was neither affected by land use nor depth and the interaction of the two.

**Table 4:** Two way analysis of variance for SOC (%), TN (%), C:N and pH (H_2_O), under different land uses, soil depths and interaction effect in Hades sub watershed

Source of variation	df	SOC		TN		C/N		pH	
		MS	Sig.	MS	Sig.	MS	Sig.	MS	Sig.
Depth	2	5.683	0.002*	0.051	0.004*	0.821	0.821	0.017	0.781
Land use	3	26.79	0.000*	0.199	0.000*	7.826	0.151	2.368	0.000*
Depth × land use	6	0.897	0.339	0.018	0.055	6.756	0.17	0.022	0.921

*The mean difference is significant at the 0.05 level

The pH of the soils under the four land use types exhibited significant (p < 0.05) variation across land uses but not within soil depth across the land uses (Table 5). At the surface layers, relatively higher pH value was recorded in the soils of the natural forest, whereas relatively lower value was recorded in the cultivated land. The soil organic carbon content was significantly different among land uses, and soil depths within a given land use (Table 5).

**Table 5:** Contents of selected soil chemical properties in relation to different land uses and soil depth (mean ± SD)

Variable	Depth (m)	Land Uses				
		NF	CA	GL	CR	Overall
pH (H_2_O)	0–0.2	7.23 (± 0.16)	6.94 (± 0.04)	6.8 (± 0.49)	6.3 (± 0.04)	6.91 (± 0.41)
	0.2–0.4	7.34 (± 0.16)	6.89 (± 0.1)	6.73 (± 0.64)	6.11 (± 0.05)	6.89 (± 0.55)
	0.4–0.6	7.35 (± 0.08)	6.89 (± 0.09)	6.85 (± 0.52)	6.33 (± 0.09)	6.94 (± 0.46)
	Overall	7.31 (± 0.15)c	6.91 (± 0.07)b	6.80 (± 0.48)b	6.23 (± 0.12)a	
OC (%)	0–0.2	6.38 (± 0.59)	3.39 (± 0.45)	2.71 (± 0.29)	1.86 (± 0.12)	4.02 (2.04)b
	0.2–0.4	4.22 (± 0.39)	2.93 (± 0.12)	2.23 (± 0.20)	1.63 (± 0.19)	3.12 (1.43)a
	0.4–0.6	2.89 (± 0.8)	1.94 (± 0.15)	1.68 (± 0.19)	1.15 (± 0.26)	2.52 (1.31)a
	Overall	4.80 (± 1.57)c	2.77 (± 0.66)b	2.15 (± 0.42)ab	1.61 (± 0.34)a	
TN (%)	0–0.2	049 (± 0.11)	0.28 (± 0.07)	0.16 (± 0.01)	0.13 (± 0.01)	0.33 (± 0.22)b
	0.2–0.4	0.36 (± 0.03)	0.21 (± 0.03)	0.17 (± 0.03)	0.1 (± 0.02)	0.23 (± 0.11)a
	0.4–0.6	0.31 (± 0.04)	0.15 (± 0.04)	0.13 (± 0.01)	0.09 (± 0.02)	0.18 (± 0.09)a
	Overall	0.38 (± 0.17)b	0.21 (± 0.07)a	0.15 (± 0.03)a	0.11 (± 0.02)a	
C:N	0–0.2	12.13 (± 0.54)	12.62 (± 3.130)	16.58 (± 1.03)	14.79 (± 0.75)	13.33 (± 2.38)
	0.2–0.4	12.46 (± 0.39)	14.44 (± 1.97)	13.43 (± 1.75)	16.15 (± 2.33)	13.93 (± 2.21)
	0.4–0.6	11.52 (± 1.11)	13.57 (± 2.92)	12.96 (± 1.35)	12.31 (± 0.24)	14.01 (± 1.95)
	Overall	13.08 (± 2.04)	13.61 (± 2.37)	13.99 (± 1.73)	15.03 (± 2.28)	

*TN* total nitrogen, *NF* natural forest, *CF* coffee agroforestry, *GL* grazing land, *CR* cropland

For each parameter, different letters indicate significant differences (p < 0.5) with respect to land uses, respectively at each depth (p < 0.05)

Across the three depths, significantly higher organic carbon content was found in the natural forest followed by the coffee agroforestry. The cultivated land, on the contrary, had comparatively lower values of soil organic carbon across its depths. In all the land uses, the soil organic carbon content significantly decreased with soil depth (Table 5). Similar to the soil organic carbon content, significantly high total nitrogen content was found in soils under natural forest followed by the coffee agroforestry (Table 5). This is also supported by the highly significant (p < 0.01) and positive correlation (r = 0.96) between total nitrogen and organic carbon content (Table 6). Furthermore, it also decreased with soil depth in all the land uses except the coffee agroforestry where it did not show any consistent trend down the soil depth. Similarly, sand, silt and clay correlated well with SOC (Table 6).

**Table 6:** Simple correlation analysis results of soil chemical and physical properties

	pH	OC	TN	Sand	Silt	Clay	BD
pH	1.00						
OC	0.81**	1.00					
TN	0.72*	0.96**	1.00				
Sand	0.41	0.70*	0.62*	1.00			
Silt	0.70*	0.64*	0.61*	0.14	1.00		
Clay	− 0.71*	− 0.89**	− 0.81**	− 0.81**	− 0.69*	1.00	
BD		− 0.09		0.12	0.08	− 0.14	1.00

*The mean difference is significant at the 0.05 level

**Significant at the 0.01 level

The C:N ratio, which is a derived parameter, did not follow the trend of soil organic carbon and total nitrogen with soil depth in all the land uses except the natural forest (Table 5). Comparatively high values of C:N ratio were recorded in soils of the cultivated and grazing lands as compared to the coffee agroforestry and natural forest soils. Across the land uses, it varied within a relatively narrow range of 11:1 to 17:1.

### Soil and biomass carbon stock

#### Soil carbon stock

Comparing the four land uses, significantly higher soil organic carbon stock across the three soil depths was encountered in the natural forest. In coffee agroforestry, organic carbon stock was significantly higher compared with crop and grazing lands at 0–20 cm and 20–40 cm soil depths. However, there was no significant difference in SOC stock of the three land uses (NF; CL; GL) at 20–40 cm soil depth (Table 7). Similarly, no significant difference in SOC stock was observed between crop and grazing lands across soil depths. The decline in soil organic carbon was higher at 40–60 cm soil depth than it was at 20–40 cm in all the land uses except the natural forest where the decline was considerable at the 20–40 cm (Table 7). In all land uses, about 38–40% of SOC was found in the 0–20 cm soil layer while 68–60% was recorded in 20–40 cm and 40–60 cm soil depths respectively. In the natural forest and coffee agroforestry, respectively about 68% and 92% of the total carbon stock was found in the soil (Table 8).

**Table 7:** Soil organic carbon stock (t ha^−1^) in relation to different land uses and soil depths (mean ± SE)

Land uses	Soil depth (cm)		
	0–20	20–40	40–60
Cropland	53.11 (± 8.42)c	48.81 (± 8.04)b	37.03 (± 8.91)b
Grazing land	57.38 (± 10.29)c	55.4 (± 2.55)b	42.34 (± 4.71)b
Coffee agroforestry	93.78 (± 6.92)b	81.07 (± 16.69)a	60.31 (± 15.31)b
Natural forest	141.34 (± 12.32)a	101.36 ± (11.26)a	103 (± 23.06)a

a, b, c means with the same letter (s) across columns and rows are not significantly different

**Table 8:** Mean vegetation and soil carbon stock (t ha^−1^) of different land uses (mean ± SE)

LU	Carbon stock in different land uses (t/ha)				Total carbon
	AGC	BGC	LC	SOC	
Natural forest	116.46 ± 17.81	23.29 ± 3.56	0.69 ± 0.08	339.19 ± 21.09	496.26 ± 11.28c
Coffee agroforestry	17.26 ± 1.9	3.43 ± 0.34	0.36 ± 0.04	249.69 ± 28.13	277.38 ± 28.58b
Grazing land	–	–		155.13 ± 11.46	155.13 ± 11.46a
Crop land	–	–	–	138 ± 0.95 ± 25.01	138.95 ± 25.0.1a

*AGC* aboveground carbon, *BGC* belowground carbon, *LC* litter carbon, *UC* undergrowth carbon, *SOC* soil organic carbon

a, b, c means with the same letter (s) across columns and rows are not significantly different

#### Deterioration index

**Fig. 3:**
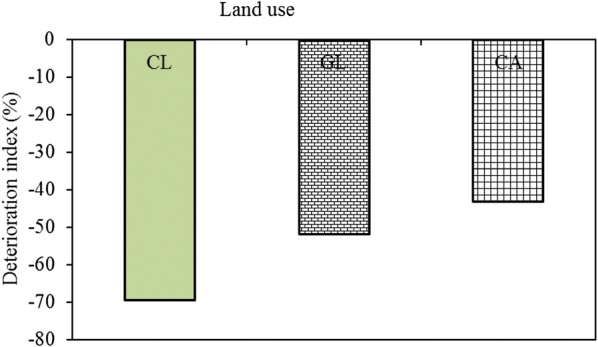
Deterioration index of major land uses as compared to the natural forest land in the sub-watershed

The deterioration index clearly shows that the conversion of the once natural forest ecosystem into managed ecosystems in the form of grazing land, coffee agroforestry, and crop land has resulted in net degradation in organic carbon and total nitrogen levels. The decline in these soil attributes was more severe in soils that were under crop cultivation for the last many years (Fig. 3). This is followed by the grazing land. However, there was relatively lower deterioration index under the coffee agroforestry.

#### Biomass carbon stock

Due to absence of trees on sample plots under crop and grazing lands, biomass measurement was only made on natural forest and coffee agroforestry land uses. The natural forest was found to have significantly higher biomass carbon stock compared with the coffee agroforestry land use. In the natural forest, 81.5% share of the biomass carbon stock was attributed to the aboveground biomass. Particularly trees with dbh ≥ 30 cm had contributed the largest carbon. The contribution of the belowground (root), litter, and undergrowth vegetation in the natural forest was 18.5% of the total biomass carbon stock. In coffee agroforestry land use, only a small number of shade trees with small diameter were encountered and measured. Accordingly, the share of aboveground biomass (shade trees and coffee shrub) was 98.29% of the biomass carbon stock of coffee agroforestry land use. The undergrowth carbon stock recorded under the natural forest was 0.38 ± 0.08 t ha^−1^, which was lower than the other carbon pools (Table 8).

## Discussion

### Soil properties

The presence of significantly more sand content in the natural forest than other land uses is probably due to the steep slope and high elevation where the forest is located. Nahusenay and Kibebew [49] also reported significantly higher sand content in soils under natural forest as compared to shrub, grazing, and cultivated lands in North-central highlands of Ethiopia. The presence of high clay content at 0–20 cm soil depth of the cultivated and grazing lands indicates the selective removal of the finer particles, such as silt and clay, by water erosion from the steep slopes where the natural forest land is located, and their subsequent accumulation in the gently sloping and low-lying parts of the study area where the grazing and cultivated lands are located. Ellerbrck and Gerke [50] pointed out that during erosion clay particles can be transported along hill slopes and form colluvic soil at the topographic depressions. In line with the findings of the current study, [51] reported accumulation of clay particles at lower soil depths.

The lower bulk density values in the natural forest could be associated with the high organic carbon content which encourages aggregation. Teshome et al. [52, 53] also reported significantly lower bulk density values in the natural forest as compared to grazing and cultivated lands in western and central highlands of Ethiopia. The inverse relationship between organic matter and bulk density was also reported by many researchers [54]. The increase in soil bulk density with soil depth might be associated with the decline in organic matter, aggregation, and root penetration in addition to the expected compacting effect of the overlying soil mass. A number of studies conducted in Ethiopia have reported a general increase in bulk density value with soil depth [54, 55]. The bulk density values of the soils under the four land use types are not likely to restrict plant growth as a consequence of excessive compaction [56].

The lower pH value in soils of the cultivated land could be due to depletion of basic cations through crop harvest and continuous use of acid-forming fertilizers, such as di-ammonium phosphate (NH_4_)_2_HPO_4_), which produces strong acids when oxidized by soil microbes [57]. The relatively higher pH values recorded at the bottom layers (40–60 cm) in some of the land uses could be related to the leaching of the basic cations from the upper layers and their subsequent deposition at the lower soil depths [58]. Following soil pH rating suggested by [59], the pH values of the studied soils fall within the range of slightly acidic in the cultivated land to neutral in the other land use types. The pH values recorded in soils under the four land uses are within the range that is considered favorable for availability of most plant nutrients, growth of plants, and activity of microorganisms [60].

The higher total nitrogen level under the natural forest could be attributed to its better organic carbon content, which is the major source of total nitrogen (essentially organic nitrogen) [60]. This is also supported by the highly significant (p < 0.01) and positive correlation (r = 0.96) between total nitrogen and organic carbon content (Table 6). Loss of considerable total nitrogen following conversion of land from forest to cultivated land was reported in many similar studies conducted elsewhere [49, 53, 61–64].

Some of the C:N ratios were outside the range that is considered normal (10–12) for arable mineral soils. As indicated by [65], ratios between 15 and 25 indicate a slow decomposition process due to scarcity of nitrogen. Accordingly, the ratios recorded in some surface and subsurface layers of the cultivated, grazing, and natural forest lands indicate the presence of slow decomposition process probably due to the presence of resistant structures and organic compounds. Strong and Mason [66] pointed out that organic matter with a high C:N ratio (> 20) locks up nitrogen as it decomposes, decreasing available nitrogen for the crop.

### Soil carbon stock

The presence of high SOC in the upper 20 cm of the soil indicates the large amount of carbon to be lost if the upper 20 cm layer of the soil is disturbed, particularly in uncultivated lands. Besides the presence of high SOC in the upper 40 cm of coffee agroforestry, grazing, and crop land uses indicate the depth to that level is as important as the surface layers for storing carbon. On the other hand, the presence of 68–60% of the organic carbon in the lower (0.2–0.4 m and 0.4–0.6 m) soil layers indicates that the deeper layers of the soil are also important pools in terms of preserving soil organic carbon for a long time. Several other studies have also reported the presence of relatively higher organic carbon values in the upper layers of soils as compared with the lower or deeper layers [34, 67–70]. The lower SOC in the crop lands could be due to repeated cultivation before sowing [10], removal of crop residues [7, 14, 71], and burning of crop residues during land preparation [11]. The current results indicate that the subsistence farming practices that have been followed by the smallholder farmers in the study area are exploitative in nature, and thus, have taken much of the soil organic carbon as compared to the natural forest. This loss of organic carbon is likely to increase emission from the soil system. A land use type that enhances the organic carbon content and minimizes emission has to be put in place in order to fight climate change and its unfavorable impacts. It is well understood that management practices have impact on the balance between input and output in a given system as well as on the rate of decomposition of organic matter [10]. Therefore, appropriate land use and management practices [12, 14] such as conservation tillage [72], no tillage and fertilization/intensive agriculture [15], and agroforestry [73] should be employed to enhance the carbon sequestration potential of croplands under subsistence low input farming.

Even though the result of the study revealed higher SOC in grazing land compared with the national average of 26.2 t ha^−1^ [74], it is believed that excessive grazing pressure and the cut and carry system commonly used in the sub watershed have resulted in lower organic matter input into the system [75, 76]. The result also agreed with the findings of [77] who indicated the effect of grazing on plant biomass through removal of palatable plants which are high quality energy sources for decomposers. Enhancing carbon stock in grazing lands can be achieved through improved grazing management such as optimizing stock number, rotational grazing, and fertilization [77]. However, the rotational grazing option is not feasible in the current context of the study area mainly due to population pressure and higher demand for agricultural land expansion. Hence, implementation of other options such as introduction of fodder species on marginal and other lands, and establishing fodder grasses on bunds are essential to minimize the grazing pressure and enhance the C sequestration potential of the grazing land.

The higher soil organic carbon stock in the natural forest might be attributed to the frequent addition of litter, the presence of network of roots, and modified microclimate, which retard decomposition rate of organic matter. The finding agrees with the findings of [78, 79] who reported higher SOC stock under forest than other land use types. Woldeamlak and Stroosnijder [80] also found high soil organic carbon in natural forest compared with grazing land, land under cultivation, and Eucalyptus plantation. Similarly, [51] reported high SOC in native forest compared with cultivated land and found a non-significant difference between native forest and grazing land. Noordwijk et al. [78, 79] also reported high SOC in disturbed forests compared with coffee based agroforestry.

According to information obtained from local agriculture office, most of the land that represents the different levels of the variable ‘land use’ used to be under natural forest cover many decades ago. Which was indeed attested by the presence of some remnants of naturally grown trees in the land under different land uses today. Hence, conversion from the natural system to human managed system has resulted in the loss of significant amount of SOC [9, 17–20, 75, 81, 82]. The declining trend in SOC could be due to repeated disturbance of the soil after conversion of forest land into other land uses [9, 11, 83].

Frequent addition of partially decomposed organic matter, presence of woody perennials, and well-built conservation structures have made coffee agroforestry to have higher SOC compared with grazing and crop lands. Reports [84] also witnessed the importance of management and biotic factors in influencing soil organic carbon at local level. The contribution of SOC to the total carbon stock of coffee agroforestry was 94.5%, which agrees with the findings of [78, 79, 85]. Nair et al. [73] indicated that time sequence studies on soil carbon are rare in agroforestry systems; hence the current study will serve as a bench mark for the study area.

The presence of SOC in lower soil depths indicates how these layers of the soil are important pools in terms of preserving soil organic carbon for a long time [86]. Unlike studies elsewhere in Ethiopia [87–91], the current study yielded high SOC in the natural forest.

### Deterioration index

The relatively lower deterioration index under the coffee agroforestry could be associated with the relatively better soil management. Studies conducted elsewhere in Ethiopia (e.g., [52]) also indicated a high deterioration index under cultivated lands as compared with other land uses. These, suggesting that most of the smallholder subsistence farming practices in Ethiopia are highly exploitative and undermine the potential of the agriculture sector to sequester carbon and help in mitigating climate change. The results also imply that other alternative land uses with appropriate management strategies (e.g. climate-smart agriculture), which enhance the carbon stock and carbon sequestration potential of the lands while minimizing emissions, should be put in place to boost the subsistence farmers’ adaptive capacity against climate change.

### Biomass carbon stock

The average AGC (116.46 t C ha^−1^) of the current finding is greater than tropical (45.45 t C ha^−1^) and regional (58.9 t C ha^−1^) estimates [43, 92]. On the other hand, all recently assessed forests in Ethiopia exhibited higher aboveground, root, and litter carbon stock compared to the finding of the current study [87–91]. The status of the forest and its composition of species, along with climatic and edaphic factors, might be responsible for the differences in aboveground, root, and litter carbon stock. Besides, the lower litter carbon in the study area could be related to the collection of twigs and branches for fuelwood by the local community. The lower carbon stock in the undergrowth vegetation of the natural forest could be due to the presence of large trees, though very small in number, whose shading effect may inhibit the growth of undergrowth vegetation. As put by [93], biomass of the understory declines as the forest age increases. The absence of undergrowth vegetation in coffee agroforestry sites is primarily due to intensive weeding and soil management works.

The coffee agroforestry had lower vegetation carbon stock compared with similar studies in Guatimala [79] and Costa Rica [85]. The lower carbon stock in the current study could be attributed to the presence of a very small number of trees with small diameter. The aboveground carbon stock in agroforestry relate with the density of coffee shrub and shade trees [85], type of shade trees used [34, 94], and size of the plant (current study). Hence, the combination of these three criteria provides reasonable outcome. Nonetheless, it is also important to increase diversity of species, most importantly woody perennials, to enhance the service [36] and productive roles [95, 96] of coffee agroforestry in the study area.

## Conclusions

The study demonstrates that even under poor management, soil has higher carbon stock compared with aboveground biomass. The subsistence farming practice followed by the smallholder farmers in the study area is exploitative in nature. As a result, it has taken much of the soil organic carbon, which was found to be relatively higher in the natural forest. This loss of organic carbon is likely to increase emission from the soil system. Also, the climax reaching vegetation and its impacts on undergrowth plants render the natural forest low carbon sequestration potential. Hence, it is necessary to implement conservation-based production systems with inclusion of woody perennials. Although there is scarcity of information on carbon stock under different land uses in Ethiopia, the present study highlights the importance of watershed level carbon stock assessment for better and carbon friendly land use decision making. The study, however, did not attempt to assess the carbon gain and loss as a function of each land use, so further study is needed to clearly map carbon sequestration potential of the sub-watershed.

## Abbreviations

AGB: above ground biomass
BD: bulk density
CA: coffee agroforestry
CL: crop land
DI: deterioration index
GHG: green house gas
GL: grazing land
NF: natural forest
OM: organic matter
SOC: soil organic carbon

## Availability of data and materials

Data is available in Springer nature.
